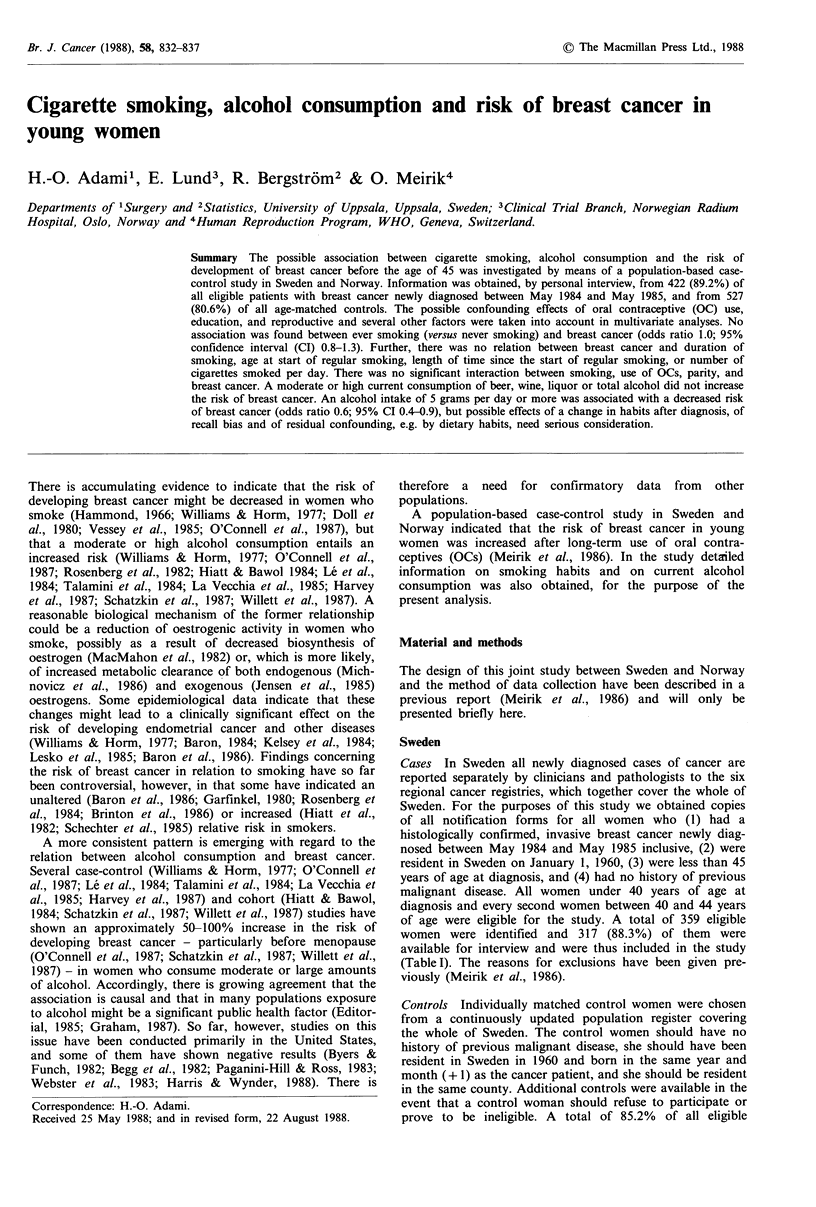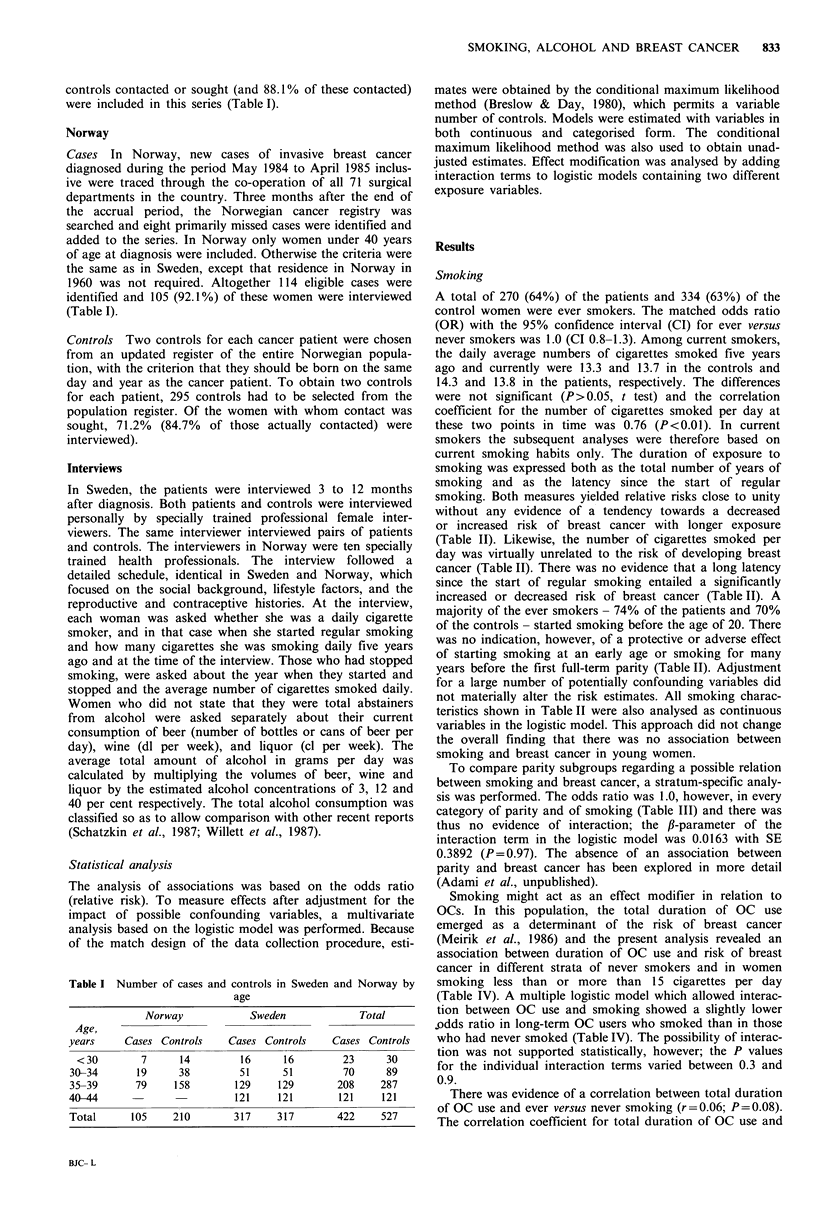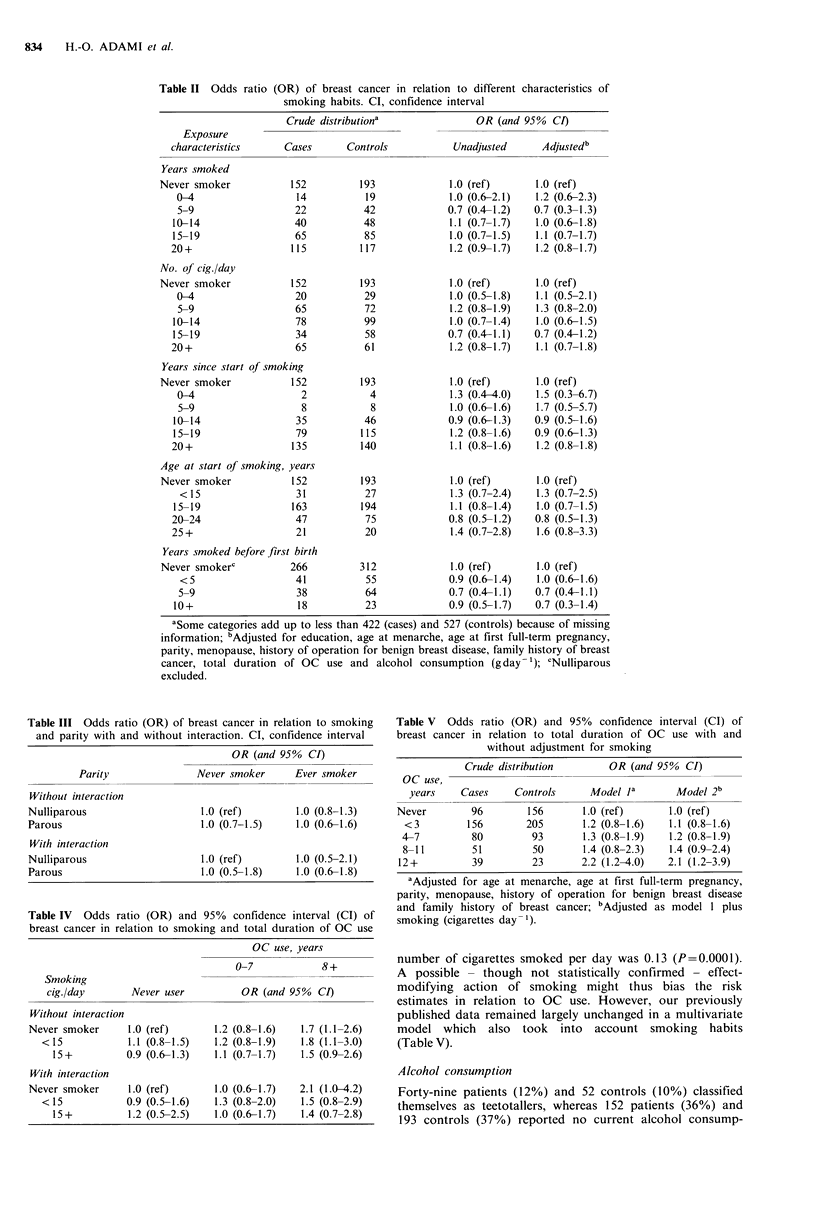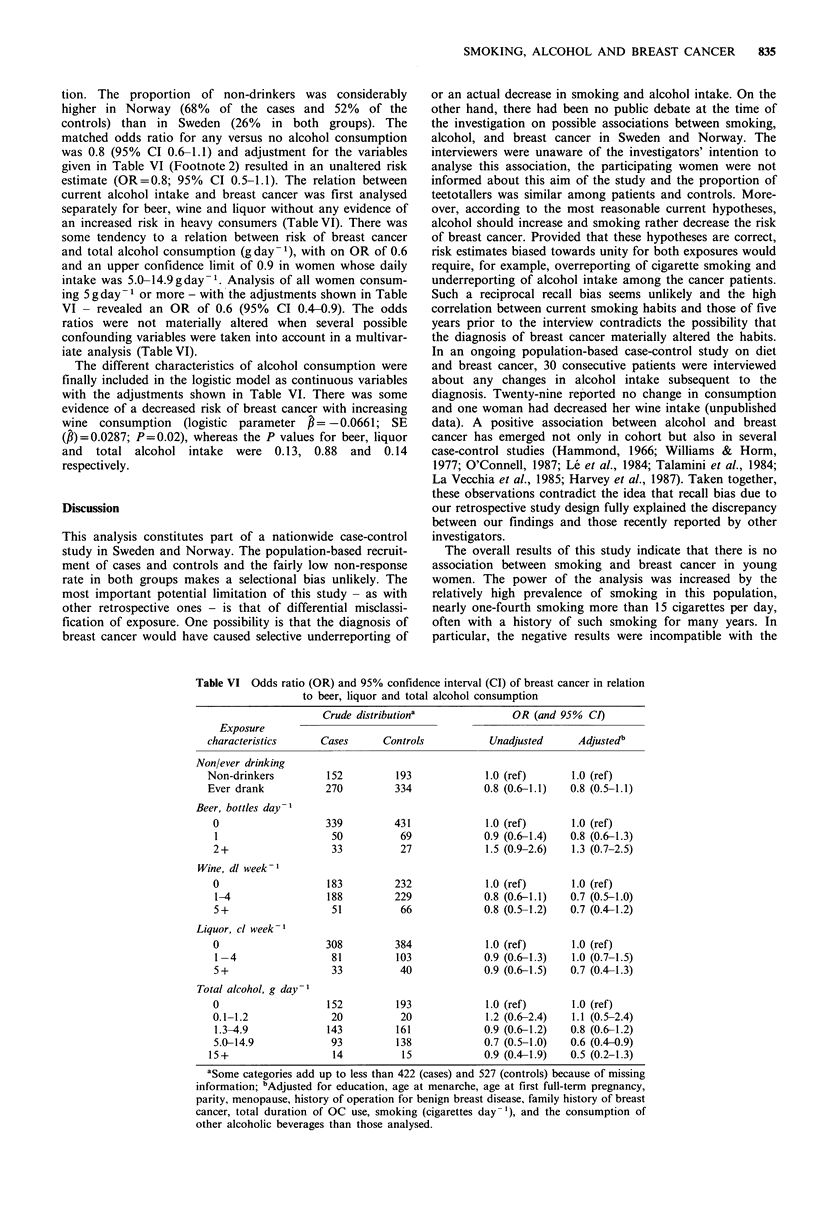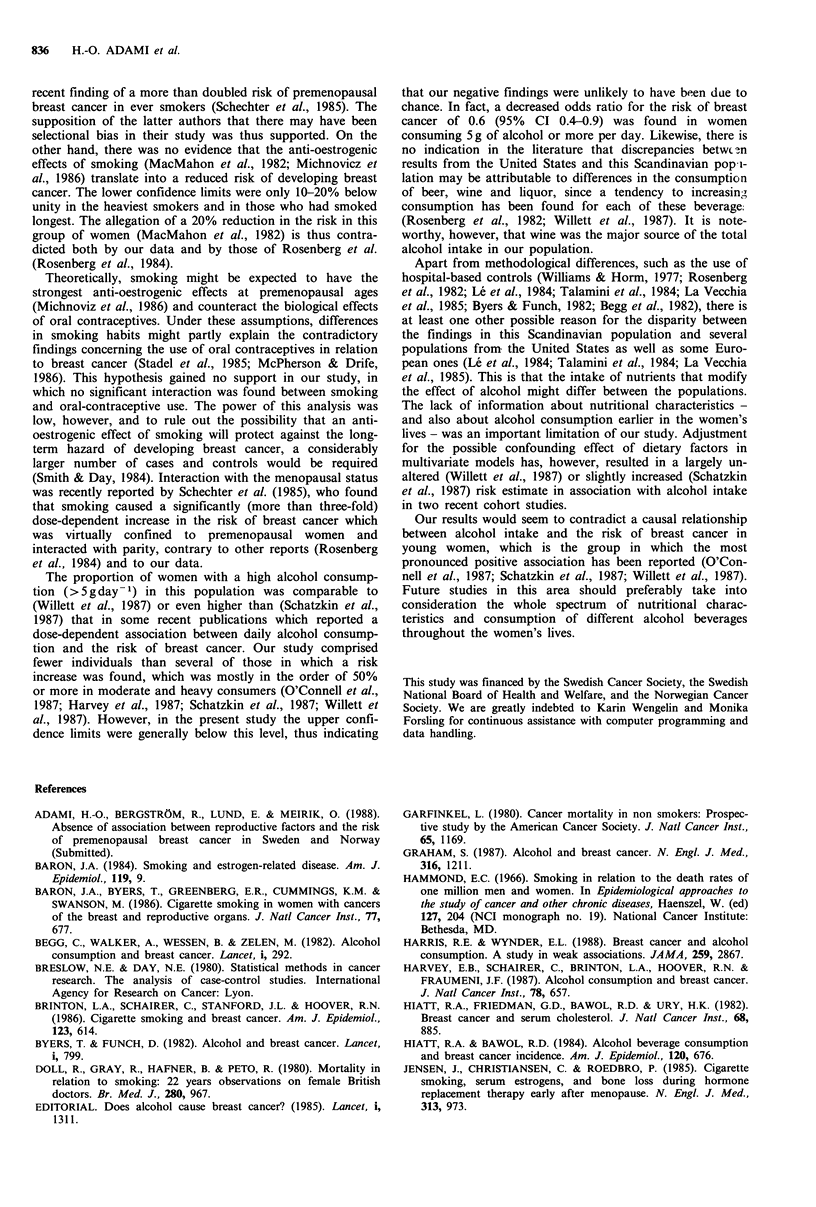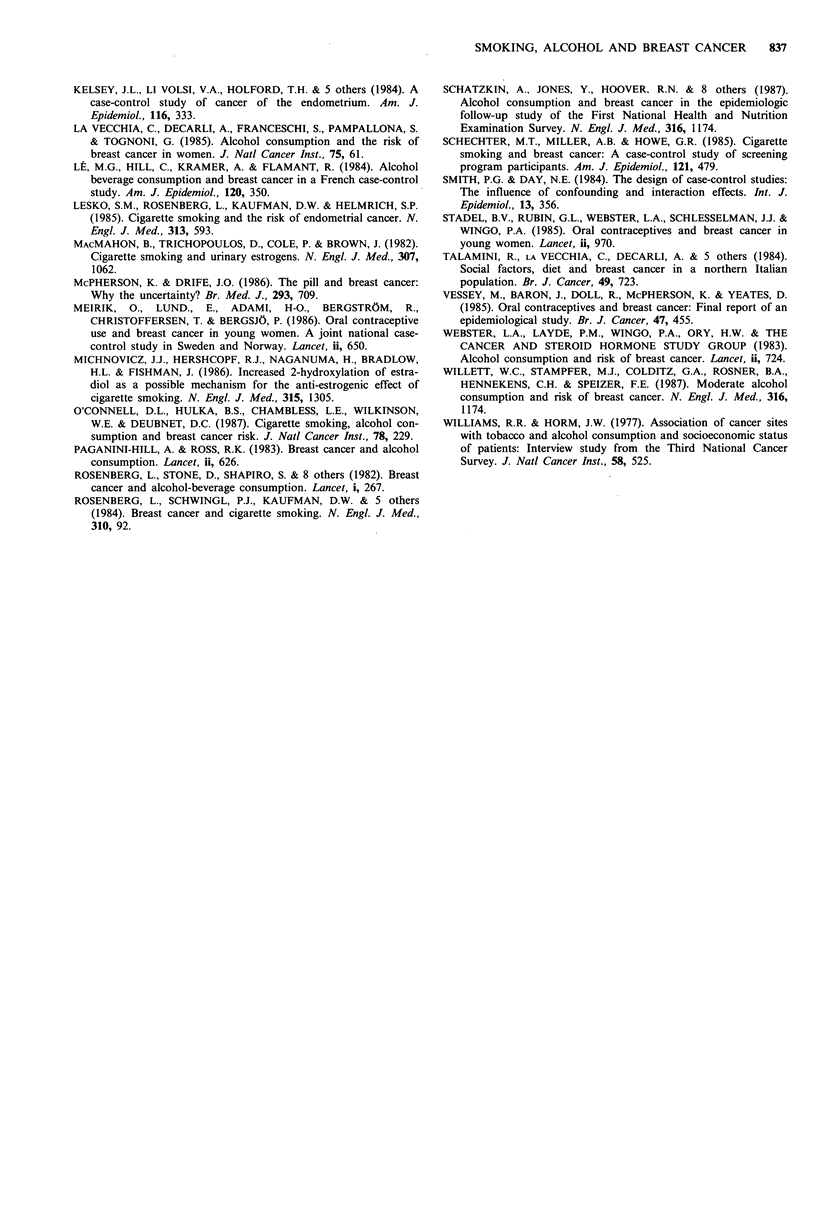# Cigarette smoking, alcohol consumption and risk of breast cancer in young women.

**DOI:** 10.1038/bjc.1988.320

**Published:** 1988-12

**Authors:** H. O. Adami, E. Lund, R. Bergström, O. Meirik

**Affiliations:** Department of Surgery, University of Uppsala, Sweden.

## Abstract

The possible association between cigarette smoking, alcohol consumption and the risk of development of breast cancer before the age of 45 was investigated by means of a population-based case-control study in Sweden and Norway. Information was obtained, by personal interview, from 422 (89.2%) of all eligible patients with breast cancer newly diagnosed between May 1984 and May 1985, and from 527 (80.6%) of all age-matched controls. The possible confounding effects of oral contraceptive (OC) use, education, and reproductive and several other factors were taken into account in multivariate analyses. No association was found between ever smoking (versus never smoking) and breast cancer (odds ratio 1.0; 95% confidence interval (CI) 0.8-1.3). Further, there was no relation between breast cancer and duration of smoking, age at start of regular smoking, length of time since the start of regular smoking, or number of cigarettes smoked per day. There was no significant interaction between smoking, use of OCs, parity, and breast cancer. A moderate or high current consumption of beer, wine, liquor or total alcohol did not increase the risk of breast cancer. An alcohol intake of 5 grams per day or more was associated with a decreased risk of breast cancer (odds ratio 0.6; 95% CI 0.4-0.9), but possible effects of a change in habits after diagnosis, of recall bias and of residual confounding, e.g. by dietary habits, need serious consideration.


*Br. J. Cancer* (1988), **58**, 832–837 © The Macmillan Press Ltd., 1988

### Cigarette smoking, alcohol consumption and risk of breast cancer in young women

H.-O. Adami[1], E. Lund[3], R. Bergström[2] & O. Meirik[4]

*Departments of [1]Surgery and [2]Statistics, University of Uppsala, Uppsala, Sweden; [3]Clinical Trial Branch, Norwegian Radium Hospital, Oslo, Norway and [4]Human Reproduction Program, WHO, Geneva, Switzerland.*

**Summary** The possible association between cigarette smoking, alcohol consumption and the risk of development of breast cancer before the age of 45 was investigated by means of a population-based case-control study in Sweden and Norway. Information was obtained, by personal interview, from 422 (89.2%) of all eligible patients with breast cancer newly diagnosed between May 1984 and May 1985, and from 527 (80.6%) of all age-matched controls. The possible confounding effects of oral contraceptive (OC) use, education, and reproductive and several other factors were taken into account in multivariate analyses. No association was found between ever smoking (*versus* never smoking) and breast cancer (odds ratio 1.0; 95% confidence interval (CI) 0.8–1.3). Further, there was no relation between breast cancer and duration of smoking, age at start of regular smoking, length of time since the start of regular smoking, or number of cigarettes smoked per day. There was no significant interaction between smoking, use of OCs, parity, and breast cancer. A moderate or high current consumption of beer, wine, liquor or total alcohol did not increase the risk of breast cancer. An alcohol intake of 5 grams per day or more was associated with a decreased risk of breast cancer (odds ratio 0.6; 95% CI 0.4–0.9), but possible effects of a change in habits after diagnosis, of recall bias and of residual confounding, e.g. by dietary habits, need serious consideration.

There is accumulating evidence to indicate that the risk of developing breast cancer might be decreased in women who smoke (Hammond, 1966; Williams & Horm, 1977; Doll *et al.*, 1980; Vessey *et al.*, 1985; O'Connell *et al.*, 1987), but that a moderate or high alcohol consumption entails an increased risk (Williams & Horm, 1977; O'Connell *et al.*, 1987; Rosenberg *et al.*, 1982; Hiatt & Bawol 1984; Lê *et al.*, 1984; Talamini *et al.*, 1984; La Vecchia *et al.*, 1985; Harvey *et al.*, 1987; Schatzkin *et al.*, 1987; Willett *et al.*, 1987). A reasonable biological mechanism of the former relationship could be a reduction of oestrogenic activity in women who smoke, possibly as a result of decreased biosynthesis of oestrogen (MacMahon *et al.*, 1982) or, which is more likely, of increased metabolic clearance of both endogenous (Michnovicz *et al.*, 1986) and exogenous (Jensen *et al.*, 1985) oestrogens. Some epidemiological data indicate that these changes might lead to a clinically significant effect on the risk of developing endometrial cancer and other diseases (Williams & Horm, 1977; Baron, 1984; Kelsey *et al.*, 1984; Lesko *et al.*, 1985; Baron *et al.*, 1986). Findings concerning the risk of breast cancer in relation to smoking have so far been controversial, however, in that some have indicated an unaltered (Baron *et al.*, 1986; Garfinkel, 1980; Rosenberg *et al.*, 1984; Brinton *et al.*, 1986) or increased (Hiatt *et al.*, 1982; Schechter *et al.*, 1985) relative risk in smokers.

A more consistent pattern is emerging with regard to the relation between alcohol consumption and breast cancer. Several case-control (Williams & Horm, 1977; O'Connell *et al.*, 1987; Lê *et al.*, 1984; Talamini *et al.*, 1984; La Vecchia *et al.*, 1985; Harvey *et al.*, 1987) and cohort (Hiatt & Bawol, 1984; Schatzkin *et al.*, 1987; Willett *et al.*, 1987) studies have shown an approximately 50–100% increase in the risk of developing breast cancer – particularly before menopause (O'Connell *et al.*, 1987; Schatzkin *et al.*, 1987; Willett *et al.*, 1987) – in women who consume moderate or large amounts of alcohol. Accordingly, there is growing agreement that the association is causal and that in many populations exposure to alcohol might be a significant public health factor (Editorial, 1985; Graham, 1987). So far, however, studies on this issue have been conducted primarily in the United States, and some of them have shown negative results (Byers & Funch, 1982; Begg *et al.*, 1982; Paganini-Hill & Ross, 1983; Webster *et al.*, 1983; Harris & Wynder, 1988). There is therefore a need for confirmatory data from other populations.

A population-based case-control study in Sweden and Norway indicated that the risk of breast cancer in young women was increased after long-term use of oral contraceptives (OCs) (Meirik *et al.*, 1986). In the study detailed information on smoking habits and on current alcohol consumption was also obtained, for the purpose of the present analysis.

### Material and methods

The design of this joint study between Sweden and Norway and the method of data collection have been described in a previous report (Meirik *et al.*, 1986) and will only be presented briefly here.

### Sweden

*Cases* In Sweden all newly diagnosed cases of cancer are reported separately by clinicians and pathologists to the six regional cancer registries, which together cover the whole of Sweden. For the purposes of this study we obtained copies of all notification forms for all women who (1) had a histologically confirmed, invasive breast cancer newly diagnosed between May 1984 and May 1985 inclusive, (2) were resident in Sweden on January 1, 1960, (3) were less than 45 years of age at diagnosis, and (4) had no history of previous malignant disease. All women under 40 years of age at diagnosis and every second women between 40 and 44 years of age were eligible for the study. A total of 359 eligible women were identified and 317 (88.3%) of them were available for interview and were thus included in the study (Table I). The reasons for exclusions have been given previously (Meirik *et al.*, 1986).

*Controls* Individually matched control women were chosen from a continuously updated population register covering the whole of Sweden. The control women should have no history of previous malignant disease, she should have been resident in Sweden in 1960 and born in the same year and month (+1) as the cancer patient, and she should be resident in the same county. Additional controls were available in the event that a control woman should refuse to participate or prove to be ineligible. A total of 85.2% of all eligible

Correspondence: H.-O. Adami.
Received 25 May 1988; and in revised form, 22 August 1988.

controls contacted or sought (and 88.1% of these contacted) were included in this series (Table I).

### Norway

*Cases* In Norway, new cases of invasive breast cancer diagnosed during the period May 1984 to April 1985 inclusive were traced through the co-operation of all 71 surgical departments in the country. Three months after the end of the accrual period, the Norwegian cancer registry was searched and eight primarily missed cases were identified and added to the series. In Norway only women under 40 years of age at diagnosis were included. Otherwise the criteria were the same as in Sweden, except that residence in Norway in 1960 was not required. Altogether 114 eligible cases were identified and 105 (92.1%) of these women were interviewed (Table I).

*Controls* Two controls for each cancer patient were chosen from an updated register of the entire Norwegian population, with the criterion that they should be born on the same day and year as the cancer patient. To obtain two controls for each patient, 295 controls had to be selected from the population register. Of the women with whom contact was sought, 71.2% (84.7% of those actually contacted) were interviewed).

### Interviews

In Sweden, the patients were interviewed 3 to 12 months after diagnosis. Both patients and controls were interviewed personally by specially trained professional female interviewers. The same interviewer interviewed pairs of patients and controls. The interviewers in Norway were ten specially trained health professionals. The interview followed a detailed schedule, identical in Sweden and Norway, which focused on the social background, lifestyle factors, and the reproductive and contraceptive histories. At the interview, each woman was asked whether she was a daily cigarette smoker, and in that case when she started regular smoking and how many cigarettes she was smoking daily five years ago and at the time of the interview. Those who had stopped smoking, were asked about the year when they started and stopped and the average number of cigarettes smoked daily. Women who did not state that they were total abstainers from alcohol were asked separately about their current consumption of beer (number of bottles or cans of beer per day), wine (dl per week), and liquor (cl per week). The average total amount of alcohol in grams per day was calculated by multiplying the volumes of beer, wine and liquor by the estimated alcohol concentrations of 3, 12 and 40 per cent respectively. The total alcohol consumption was classified so as to allow comparison with other recent reports (Schatzkin *et al*., 1987; Willett *et al*., 1987).

### *Statistical analysis*

The analysis of associations was based on the odds ratio (relative risk). To measure effects after adjustment for the impact of possible confounding variables, a multivariate analysis based on the logistic model was performed. Because of the match design of the data collection procedure, estimates were obtained by the conditional maximum likelihood method (Breslow & Day, 1980), which permits a variable number of controls. Models were estimated with variables in both continuous and categorised form. The conditional maximum likelihood method was also used to obtain unadjusted estimates. Effect modification was analysed by adding interaction terms to logistic models containing two different exposure variables.

**Table I** Number of cases and controls in Sweden and Norway by age

| Age, years | *Norway* Cases | *Norway* Controls | *Sweden* Cases | *Sweden* Controls | *Total* Cases | *Total* Controls |
|---|---|---|---|---|---|---|
| <30 | 7 | 14 | 16 | 16 | 23 | 30 |
| 30–34 | 19 | 38 | 51 | 51 | 70 | 89 |
| 35–39 | 79 | 158 | 129 | 129 | 208 | 287 |
| 40–44 | — | — | 121 | 121 | 121 | 121 |
| Total | 105 | 210 | 317 | 317 | 422 | 527 |

### Results

### *Smoking*

A total of 270 (64%) of the patients and 334 (63%) of the control women were ever smokers. The matched odds ratio (OR) with the 95% confidence interval (CI) for ever *versus* never smokers was 1.0 (CI 0.8–1.3). Among current smokers, the daily average numbers of cigarettes smoked five years ago and currently were 13.3 and 13.7 in the controls and 14.3 and 13.8 in the patients, respectively. The differences were not significant ($P>0.05$, *t* test) and the correlation coefficient for the number of cigarettes smoked per day at these two points in time was 0.76 ($P<0.01$). In current smokers the subsequent analyses were therefore based on current smoking habits only. The duration of exposure to smoking was expressed both as the total number of years of smoking and as the latency since the start of regular smoking. Both measures yielded relative risks close to unity without any evidence of a tendency towards a decreased or increased risk of breast cancer with longer exposure (Table II). Likewise, the number of cigarettes smoked per day was virtually unrelated to the risk of developing breast cancer (Table II). There was no evidence that a long latency since the start of regular smoking entailed a significantly increased or decreased risk of breast cancer (Table II). A majority of the ever smokers – 74% of the patients and 70% of the controls – started smoking before the age of 20. There was no indication, however, of a protective or adverse effect of starting smoking at an early age or smoking for many years before the first full-term parity (Table II). Adjustment for a large number of potentially confounding variables did not materially alter the risk estimates. All smoking characteristics shown in Table II were also analysed as continuous variables in the logistic model. This approach did not change the overall finding that there was no association between smoking and breast cancer in young women.

To compare parity subgroups regarding a possible relation between smoking and breast cancer, a stratum-specific analysis was performed. The odds ratio was 1.0, however, in every category of parity and of smoking (Table III) and there was thus no evidence of interaction; the $\beta$-parameter of the interaction term in the logistic model was 0.0163 with SE 0.3892 ($P=0.97$). The absence of an association between parity and breast cancer has been explored in more detail (Adami *et al*., unpublished).

Smoking might act as an effect modifier in relation to OCs. In this population, the total duration of OC use emerged as a determinant of the risk of breast cancer (Meirik *et al*., 1986) and the present analysis revealed an association between duration of OC use and risk of breast cancer in different strata of never smokers and in women smoking less than or more than 15 cigarettes per day (Table IV). A multiple logistic model which allowed interaction between OC use and smoking showed a slightly lower odds ratio in long-term OC users who smoked than in those who had never smoked (Table IV). The possibility of interaction was not supported statistically, however; the *P* values for the individual interaction terms varied between 0.3 and 0.9.

There was evidence of a correlation between total duration of OC use and ever *versus* never smoking ($r=0.06$; $P=0.08$). The correlation coefficient for total duration of OC use and

**Table II** Odds ratio (OR) of breast cancer in relation to different characteristics of smoking habits. CI, confidence interval

| Exposure characteristics | Crude distribution[a] | | OR (and 95% CI) | |
|---|---|---|---|---|
| | Cases | Controls | Unadjusted | Adjusted[b] |
| *Years smoked* | | | | |
| Never smoker | 152 | 193 | 1.0 (ref) | 1.0 (ref) |
| 0–4 | 14 | 19 | 1.0 (0.6–2.1) | 1.2 (0.6–2.3) |
| 5–9 | 22 | 42 | 0.7 (0.4–1.2) | 0.7 (0.3–1.3) |
| 10–14 | 40 | 48 | 1.1 (0.7–1.7) | 1.0 (0.6–1.8) |
| 15–19 | 65 | 85 | 1.0 (0.7–1.5) | 1.1 (0.7–1.7) |
| 20+ | 115 | 117 | 1.2 (0.9–1.7) | 1.2 (0.8–1.7) |
| *No. of cig./day* | | | | |
| Never smoker | 152 | 193 | 1.0 (ref) | 1.0 (ref) |
| 0–4 | 20 | 29 | 1.0 (0.5–1.8) | 1.1 (0.5–2.1) |
| 5–9 | 65 | 72 | 1.2 (0.8–1.9) | 1.3 (0.8–2.0) |
| 10–14 | 78 | 99 | 1.0 (0.7–1.4) | 1.0 (0.6–1.5) |
| 15–19 | 34 | 58 | 0.7 (0.4–1.1) | 0.7 (0.4–1.2) |
| 20+ | 65 | 61 | 1.2 (0.8–1.7) | 1.1 (0.7–1.8) |
| *Years since start of smoking* | | | | |
| Never smoker | 152 | 193 | 1.0 (ref) | 1.0 (ref) |
| 0–4 | 2 | 4 | 1.3 (0.4–4.0) | 1.5 (0.3–6.7) |
| 5–9 | 8 | 8 | 1.0 (0.6–1.6) | 1.7 (0.5–5.7) |
| 10–14 | 35 | 46 | 0.9 (0.6–1.3) | 0.9 (0.5–1.6) |
| 15–19 | 79 | 115 | 1.2 (0.8–1.6) | 0.9 (0.6–1.3) |
| 20+ | 135 | 140 | 1.1 (0.8–1.6) | 1.2 (0.8–1.8) |
| *Age at start of smoking, years* | | | | |
| Never smoker | 152 | 193 | 1.0 (ref) | 1.0 (ref) |
| <15 | 31 | 27 | 1.3 (0.7–2.4) | 1.3 (0.7–2.5) |
| 15–19 | 163 | 194 | 1.1 (0.8–1.4) | 1.0 (0.7–1.5) |
| 20–24 | 47 | 75 | 0.8 (0.5–1.2) | 0.8 (0.5–1.3) |
| 25+ | 21 | 20 | 1.4 (0.7–2.8) | 1.6 (0.8–3.3) |
| *Years smoked before first birth* | | | | |
| Never smoker[c] | 266 | 312 | 1.0 (ref) | 1.0 (ref) |
| <5 | 41 | 55 | 0.9 (0.6–1.4) | 1.0 (0.6–1.6) |
| 5–9 | 38 | 64 | 0.7 (0.4–1.1) | 0.7 (0.4–1.1) |
| 10+ | 18 | 23 | 0.9 (0.5–1.7) | 0.7 (0.3–1.4) |

[a]Some categories add up to less than 422 (cases) and 527 (controls) because of missing information; [b]Adjusted for education, age at menarche, age at first full-term pregnancy, parity, menopause, history of operation for benign breast disease, family history of breast cancer, total duration of OC use and alcohol consumption ($g day^{-1}$); [c]Nulliparous excluded.

**Table III** Odds ratio (OR) of breast cancer in relation to smoking and parity with and without interaction. CI, confidence interval

| Parity | OR (and 95% CI) | |
|---|---|---|
| | Never smoker | Ever smoker |
| *Without interaction* | | |
| Nulliparous | 1.0 (ref) | 1.0 (0.8–1.3) |
| Parous | 1.0 (0.7–1.5) | 1.0 (0.6–1.6) |
| *With interaction* | | |
| Nulliparous | 1.0 (ref) | 1.0 (0.5–2.1) |
| Parous | 1.0 (0.5–1.8) | 1.0 (0.6–1.8) |

**Table IV** Odds ratio (OR) and 95% confidence interval (CI) of breast cancer in relation to smoking and total duration of OC use

| Smoking cig./day | Never user | OC use, years 0–7 | 8+ |
|---|---|---|---|
| | OR (and 95% CI) | | |
| *Without interaction* | | | |
| Never smoker | 1.0 (ref) | 1.2 (0.8–1.6) | 1.7 (1.1–2.6) |
| <15 | 1.1 (0.8–1.5) | 1.2 (0.8–1.9) | 1.8 (1.1–3.0) |
| 15+ | 0.9 (0.6–1.3) | 1.1 (0.7–1.7) | 1.5 (0.9–2.6) |
| *With interaction* | | | |
| Never smoker | 1.0 (ref) | 1.0 (0.6–1.7) | 2.1 (1.0–4.2) |
| <15 | 0.9 (0.5–1.6) | 1.3 (0.8–2.0) | 1.5 (0.8–2.9) |
| 15+ | 1.2 (0.5–2.5) | 1.0 (0.6–1.7) | 1.4 (0.7–2.8) |

**Table V** Odds ratio (OR) and 95% confidence interval (CI) of breast cancer in relation to total duration of OC use with and without adjustment for smoking

| OC use, years | Crude distribution | | OR (and 95% CI) | |
|---|---|---|---|---|
| | Cases | Controls | Model 1[a] | Model 2[b] |
| Never | 96 | 156 | 1.0 (ref) | 1.0 (ref) |
| <3 | 156 | 205 | 1.2 (0.8–1.6) | 1.1 (0.8–1.6) |
| 4–7 | 80 | 93 | 1.3 (0.8–1.9) | 1.2 (0.8–1.9) |
| 8–11 | 51 | 50 | 1.4 (0.8–2.3) | 1.4 (0.9–2.4) |
| 12+ | 39 | 23 | 2.2 (1.2–4.0) | 2.1 (1.2–3.9) |

[a]Adjusted for age at menarche, age at first full-term pregnancy, parity, menopause, history of operation for benign breast disease and family history of breast cancer; [b]Adjusted as model 1 plus smoking (cigarettes $day^{-1}$).

number of cigarettes smoked per day was 0.13 ($P = 0.0001$). A possible – though not statistically confirmed – effect-modifying action of smoking might thus bias the risk estimates in relation to OC use. However, our previously published data remained largely unchanged in a multivariate model which also took into account smoking habits (Table V).

### *Alcohol consumption*

Forty-nine patients (12%) and 52 controls (10%) classified themselves as teetotallers, whereas 152 patients (36%) and 193 controls (37%) reported no current alcohol consumption. The proportion of non-drinkers was considerably higher in Norway (68% of the cases and 52% of the controls) than in Sweden (26% in both groups). The matched odds ratio for any versus no alcohol consumption was 0.8 (95% CI 0.6–1.1) and adjustment for the variables given in Table VI (Footnote 2) resulted in an unaltered risk estimate (OR = 0.8; 95% CI 0.5–1.1). The relation between current alcohol intake and breast cancer was first analysed separately for beer, wine and liquor without any evidence of an increased risk in heavy consumers (Table VI). There was some tendency to a relation between risk of breast cancer and total alcohol consumption ($g\,day^{-1}$), with on OR of 0.6 and an upper confidence limit of 0.9 in women whose daily intake was 5.0–14.9 $g\,day^{-1}$. Analysis of all women consuming 5 $g\,day^{-1}$ or more – with the adjustments shown in Table VI – revealed an OR of 0.6 (95% CI 0.4–0.9). The odds ratios were not materially altered when several possible confounding variables were taken into account in a multivariate analysis (Table VI).

The different characteristics of alcohol consumption were finally included in the logistic model as continuous variables with the adjustments shown in Table VI. There was some evidence of a decreased risk of breast cancer with increasing wine consumption (logistic parameter $\hat{\beta} = -0.0661$; SE $(\hat{\beta}) = 0.0287$; $P = 0.02$), whereas the $P$ values for beer, liquor and total alcohol intake were 0.13, 0.88 and 0.14 respectively.

### Discussion

This analysis constitutes part of a nationwide case-control study in Sweden and Norway. The population-based recruitment of cases and controls and the fairly low non-response rate in both groups makes a selectional bias unlikely. The most important potential limitation of this study – as with other retrospective ones – is that of differential misclassification of exposure. One possibility is that the diagnosis of breast cancer would have caused selective underreporting of or an actual decrease in smoking and alcohol intake. On the other hand, there had been no public debate at the time of the investigation on possible associations between smoking, alcohol, and breast cancer in Sweden and Norway. The interviewers were unaware of the investigators' intention to analyse this association, the participating women were not informed about this aim of the study and the proportion of teetotallers was similar among patients and controls. Moreover, according to the most reasonable current hypotheses, alcohol should increase and smoking rather decrease the risk of breast cancer. Provided that these hypotheses are correct, risk estimates biased towards unity for both exposures would require, for example, overreporting of cigarette smoking and underreporting of alcohol intake among the cancer patients. Such a reciprocal recall bias seems unlikely and the high correlation between current smoking habits and those of five years prior to the interview contradicts the possibility that the diagnosis of breast cancer materially altered the habits. In an ongoing population-based case-control study on diet and breast cancer, 30 consecutive patients were interviewed about any changes in alcohol intake subsequent to the diagnosis. Twenty-nine reported no change in consumption and one woman had decreased her wine intake (unpublished data). A positive association between alcohol and breast cancer has emerged not only in cohort but also in several case-control studies (Hammond, 1966; Williams & Horm, 1977; O'Connell, 1987; Lê *et al.*, 1984; Talamini *et al.*, 1984; La Vecchia *et al.*, 1985; Harvey *et al.*, 1987). Taken together, these observations contradict the idea that recall bias due to our retrospective study design fully explained the discrepancy between our findings and those recently reported by other investigators.

The overall results of this study indicate that there is no association between smoking and breast cancer in young women. The power of the analysis was increased by the relatively high prevalence of smoking in this population, nearly one-fourth smoking more than 15 cigarettes per day, often with a history of such smoking for many years. In particular, the negative results were incompatible with the

**Table VI** Odds ratio (OR) and 95% confidence interval (CI) of breast cancer in relation to beer, liquor and total alcohol consumption

| *Exposure characteristics* | *Crude distribution*[a] | | *OR (and 95% CI)* | |
|---|---|---|---|---|
| | *Cases* | *Controls* | *Unadjusted* | *Adjusted*[b] |
| *Non/ever drinking* | | | | |
| Non-drinkers | 152 | 193 | 1.0 (ref) | 1.0 (ref) |
| Ever drank | 270 | 334 | 0.8 (0.6–1.1) | 0.8 (0.5–1.1) |
| *Beer, bottles day*$^{-1}$ | | | | |
| 0 | 339 | 431 | 1.0 (ref) | 1.0 (ref) |
| 1 | 50 | 69 | 0.9 (0.6–1.4) | 0.8 (0.6–1.3) |
| 2+ | 33 | 27 | 1.5 (0.9–2.6) | 1.3 (0.7–2.5) |
| *Wine, dl week*$^{-1}$ | | | | |
| 0 | 183 | 232 | 1.0 (ref) | 1.0 (ref) |
| 1–4 | 188 | 229 | 0.8 (0.6–1.1) | 0.7 (0.5–1.0) |
| 5+ | 51 | 66 | 0.8 (0.5–1.2) | 0.7 (0.4–1.2) |
| *Liquor, cl week*$^{-1}$ | | | | |
| 0 | 308 | 384 | 1.0 (ref) | 1.0 (ref) |
| 1–4 | 81 | 103 | 0.9 (0.6–1.3) | 1.0 (0.7–1.5) |
| 5+ | 33 | 40 | 0.9 (0.6–1.5) | 0.7 (0.4–1.3) |
| *Total alcohol, g day*$^{-1}$ | | | | |
| 0 | 152 | 193 | 1.0 (ref) | 1.0 (ref) |
| 0.1–1.2 | 20 | 20 | 1.2 (0.6–2.4) | 1.1 (0.5–2.4) |
| 1.3–4.9 | 143 | 161 | 0.9 (0.6–1.2) | 0.8 (0.6–1.2) |
| 5.0–14.9 | 93 | 138 | 0.7 (0.5–1.0) | 0.6 (0.4–0.9) |
| 15+ | 14 | 15 | 0.9 (0.4–1.9) | 0.5 (0.2–1.3) |

[a]Some categories add up to less than 422 (cases) and 527 (controls) because of missing information; [b]Adjusted for education, age at menarche, age at first full-term pregnancy, parity, menopause, history of operation for benign breast disease, family history of breast cancer, total duration of OC use, smoking (cigarettes day$^{-1}$), and the consumption of other alcoholic beverages than those analysed.

recent finding of a more than doubled risk of premenopausal breast cancer in ever smokers (Schechter *et al.*, 1985). The supposition of the latter authors that there may have been selectional bias in their study was thus supported. On the other hand, there was no evidence that the anti-oestrogenic effects of smoking (MacMahon *et al.*, 1982; Michnovicz *et al.*, 1986) translate into a reduced risk of developing breast cancer. The lower confidence limits were only 10–20% below unity in the heaviest smokers and in those who had smoked longest. The allegation of a 20% reduction in the risk in this group of women (MacMahon *et al.*, 1982) is thus contradicted both by our data and by those of Rosenberg *et al.* (Rosenberg *et al.*, 1984).

Theoretically, smoking might be expected to have the strongest anti-oestrogenic effects at premenopausal ages (Michnoviz *et al.*, 1986) and counteract the biological effects of oral contraceptives. Under these assumptions, differences in smoking habits might partly explain the contradictory findings concerning the use of oral contraceptives in relation to breast cancer (Stadel *et al.*, 1985; McPherson & Drife, 1986). This hypothesis gained no support in our study, in which no significant interaction was found between smoking and oral-contraceptive use. The power of this analysis was low, however, and to rule out the possibility that an anti-oestrogenic effect of smoking will protect against the long-term hazard of developing breast cancer, a considerably larger number of cases and controls would be required (Smith & Day, 1984). Interaction with the menopausal status was recently reported by Schechter *et al.* (1985), who found that smoking caused a significantly (more than three-fold) dose-dependent increase in the risk of breast cancer which was virtually confined to premenopausal women and interacted with parity, contrary to other reports (Rosenberg *et al.*, 1984) and to our data.

The proportion of women with a high alcohol consumption ($>5\,g\,day^{-1}$) in this population was comparable to (Willett *et al.*, 1987) or even higher than (Schatzkin *et al.*, 1987) that in some recent publications which reported a dose-dependent association between daily alcohol consumption and the risk of breast cancer. Our study comprised fewer individuals than several of those in which a risk increase was found, which was mostly in the order of 50% or more in moderate and heavy consumers (O'Connell *et al.*, 1987; Harvey *et al.*, 1987; Schatzkin *et al.*, 1987; Willett *et al.*, 1987). However, in the present study the upper confidence limits were generally below this level, thus indicating that our negative findings were unlikely to have been due to chance. In fact, a decreased odds ratio for the risk of breast cancer of 0.6 (95% CI 0.4–0.9) was found in women consuming 5 g of alcohol or more per day. Likewise, there is no indication in the literature that discrepancies between results from the United States and this Scandinavian population may be attributable to differences in the consumption of beer, wine and liquor, since a tendency to increasing consumption has been found for each of these beverages (Rosenberg *et al.*, 1982; Willett *et al.*, 1987). It is noteworthy, however, that wine was the major source of the total alcohol intake in our population.

Apart from methodological differences, such as the use of hospital-based controls (Williams & Horm, 1977; Rosenberg *et al.*, 1982; Lê *et al.*, 1984; Talamini *et al.*, 1984; La Vecchia *et al.*, 1985; Byers & Funch, 1982; Begg *et al.*, 1982), there is at least one other possible reason for the disparity between the findings in this Scandinavian population and several populations from the United States as well as some European ones (Lê *et al.*, 1984; Talamini *et al.*, 1984; La Vecchia *et al.*, 1985). This is that the intake of nutrients that modify the effect of alcohol might differ between the populations. The lack of information about nutritional characteristics – and also about alcohol consumption earlier in the women's lives – was an important limitation of our study. Adjustment for the possible confounding effect of dietary factors in multivariate models has, however, resulted in a largely unaltered (Willett *et al.*, 1987) or slightly increased (Schatzkin *et al.*, 1987) risk estimate in association with alcohol intake in two recent cohort studies.

Our results would seem to contradict a causal relationship between alcohol intake and the risk of breast cancer in young women, which is the group in which the most pronounced positive association has been reported (O'Connell *et al.*, 1987; Schatzkin *et al.*, 1987; Willett *et al.*, 1987). Future studies in this area should preferably take into consideration the whole spectrum of nutritional characteristics and consumption of different alcohol beverages throughout the women's lives.

This study was financed by the Swedish Cancer Society, the Swedish National Board of Health and Welfare, and the Norwegian Cancer Society. We are greatly indebted to Karin Wengelin and Monika Forsling for continuous assistance with computer programming and data handling.

### References

ADAMI, H.-O., BERGSTRÖM, R., LUND, E. & MEIRIK, O. (1988). Absence of association between reproductive factors and the risk of premenopausal breast cancer in Sweden and Norway (Submitted).

BARON, J.A. (1984). Smoking and estrogen-related disease. *Am. J. Epidemiol.*, **119**, 9.

BARON, J.A., BYERS, T., GREENBERG, E.R., CUMMINGS, K.M. & SWANSON, M. (1986). Cigarette smoking in women with cancers of the breast and reproductive organs. *J. Natl Cancer Inst.*, **77**, 677.

BEGG, C., WALKER, A., WESSEN, B. & ZELEN, M. (1982). Alcohol consumption and breast cancer. *Lancet*, **i**, 292.

BRESLOW, N.E. & DAY, N.E. (1980). Statistical methods in cancer research. The analysis of case-control studies. International Agency for Research on Cancer: Lyon.

BRINTON, L.A., SCHAIRER, C., STANFORD, J.L. & HOOVER, R.N. (1986). Cigarette smoking and breast cancer. *Am. J. Epidemiol.*, **123**, 614.

BYERS, T. & FUNCH, D. (1982). Alcohol and breast cancer. *Lancet*, **i**, 799.

DOLL, R., GRAY, R., HAFNER, B. & PETO, R. (1980). Mortality in relation to smoking: 22 years observations on female British doctors. *Br. Med. J.*, **280**, 967.

EDITORIAL. Does alcohol cause breast cancer? (1985). *Lancet*, **i**, 1311.

GARFINKEL, L. (1980). Cancer mortality in non smokers: Prospective study by the American Cancer Society. *J. Natl Cancer Inst.*, **65**, 1169.

GRAHAM, S. (1987). Alcohol and breast cancer. *N. Engl. J. Med.*, **316**, 1211.

HAMMOND, E.C. (1966). Smoking in relation to the death rates of one million men and women. In *Epidemiological approaches to the study of cancer and other chronic diseases*, Haenszel, W. (ed) **127**, 204 (NCI monograph no. 19). National Cancer Institute: Bethesda, MD.

HARRIS, R.E. & WYNDER, E.L. (1988). Breast cancer and alcohol consumption. A study in weak associations. *JAMA*, **259**, 2867.

HARVEY, E.B., SCHAIRER, C., BRINTON, L.A., HOOVER, R.N. & FRAUMENI, J.F. (1987). Alcohol consumption and breast cancer. *J. Natl Cancer Inst.*, **78**, 657.

HIATT, R.A., FRIEDMAN, G.D., BAWOL, R.D. & URY, H.K. (1982). Breast cancer and serum cholesterol. *J. Natl Cancer Inst.*, **68**, 885.

HIATT, R.A. & BAWOL, R.D. (1984). Alcohol beverage consumption and breast cancer incidence. *Am. J. Epidemiol.*, **120**, 676.

JENSEN, J., CHRISTIANSEN, C. & ROEDBRO, P. (1985). Cigarette smoking, serum estrogens, and bone loss during hormone replacement therapy early after menopause. *N. Engl. J. Med.*, **313**, 973.

KELSEY, J.L., LI VOLSI, V.A., HOLFORD, T.H. & 5 others (1984). A case-control study of cancer of the endometrium. *Am. J. Epidemiol.*, **116,** 333.

LA VECCHIA, C., DECARLI, A., FRANCESCHI, S., PAMPALLONA, S. & TOGNONI, G. (1985). Alcohol consumption and the risk of breast cancer in women. *J. Natl Cancer Inst.*, **75,** 61.

LÉ, M.G., HILL, C., KRAMER, A. & FLAMANT, R. (1984). Alcohol beverage consumption and breast cancer in a French case-control study. *Am. J. Epidemiol.*, **120,** 350.

LESKO, S.M., ROSENBERG, L., KAUFMAN, D.W. & HELMRICH, S.P. (1985). Cigarette smoking and the risk of endometrial cancer. *N. Engl. J. Med.*, **313,** 593.

MACMAHON, B., TRICHOPOULOS, D., COLE, P. & BROWN, J. (1982). Cigarette smoking and urinary estrogens. *N. Engl. J. Med.*, **307,** 1062.

MCPHERSON, K. & DRIFE, J.O. (1986). The pill and breast cancer: Why the uncertainty? *Br. Med. J.*, **293,** 709.

MEIRIK, O., LUND., E., ADAMI, H-O., BERGSTRÖM, R., CHRISTOFFERSEN, T. & BERGSJÖ, P. (1986). Oral contraceptive use and breast cancer in young women. A joint national case-control study in Sweden and Norway. *Lancet*, **ii,** 650.

MICHNOVICZ, J.J., HERSHCOPF, R.J., NAGANUMA, H., BRADLOW, H.L. & FISHMAN, J. (1986). Increased 2-hydroxylation of estradiol as a possible mechanism for the anti-estrogenic effect of cigarette smoking. *N. Engl. J. Med.*, **315,** 1305.

O'CONNELL, D.L., HULKA, B.S., CHAMBLESS, L.E., WILKINSON, W.E. & DEUBNET, D.C. (1987). Cigarette smoking, alcohol consumption and breast cancer risk. *J. Natl Cancer Inst.*, **78,** 229.

PAGANINI-HILL, A. & ROSS, R.K. (1983). Breast cancer and alcohol consumption. *Lancet*, **ii,** 626.

ROSENBERG, L., STONE, D., SHAPIRO, S. & 8 others (1982). Breast cancer and alcohol-beverage consumption. *Lancet*, **i,** 267.

ROSENBERG, L., SCHWINGL, P.J., KAUFMAN, D.W. & 5 others (1984). Breast cancer and cigarette smoking. *N. Engl. J. Med.*, **310,** 92.

SCHATZKIN, A., JONES, Y., HOOVER, R.N. & 8 others (1987). Alcohol consumption and breast cancer in the epidemiologic follow-up study of the First National Health and Nutrition Examination Survey. *N. Engl. J. Med.*, **316,** 1174.

SCHECHTER, M.T., MILLER, A.B. & HOWE, G.R. (1985). Cigarette smoking and breast cancer: A case-control study of screening program participants. *Am. J. Epidemiol.*, **121,** 479.

SMITH, P.G. & DAY, N.E. (1984). The design of case-control studies: The influence of confounding and interaction effects. *Int. J. Epidemiol.*, **13,** 356.

STADEL, B.V., RUBIN, G.L., WEBSTER, L.A., SCHLESSELMAN, J.J. & WINGO, P.A. (1985). Oral contraceptives and breast cancer in young women. *Lancet*, **ii,** 970.

TALAMINI, R., LA VECCHIA, C., DECARLI, A. & 5 others (1984). Social factors, diet and breast cancer in a northern Italian population. *Br. J. Cancer*, **49,** 723.

VESSEY, M., BARON, J., DOLL, R., MCPHERSON, K. & YEATES, D. (1985). Oral contraceptives and breast cancer: Final report of an epidemiological study. *Br. J. Cancer*, **47,** 455.

WEBSTER, L.A., LAYDE, P.M., WINGO, P.A., ORY, H.W. & THE CANCER AND STEROID HORMONE STUDY GROUP (1983). Alcohol consumption and risk of breast cancer. *Lancet*, **ii,** 724.

WILLETT, W.C., STAMPFER, M.J., COLDITZ, G.A., ROSNER, B.A., HENNEKENS, C.H. & SPEIZER, F.E. (1987). Moderate alcohol consumption and risk of breast cancer. *N. Engl. J. Med.*, **316,** 1174.

WILLIAMS, R.R. & HORM, J.W. (1977). Association of cancer sites with tobacco and alcohol consumption and socioeconomic status of patients: Interview study from the Third National Cancer Survey. *J. Natl Cancer Inst.*, **58,** 525.